# B cell targeted therapies in inflammatory autoimmune disease of the central nervous system

**DOI:** 10.3389/fimmu.2023.1129906

**Published:** 2023-03-09

**Authors:** Moritz J. Furman, Sven G. Meuth, Philipp Albrecht, Michael Dietrich, Heike Blum, Jan Mares, Ron Milo, Hans-Peter Hartung

**Affiliations:** ^1^ Department of Neurology, Heinrich-Heine University Düsseldorf, Medical Faculty, Düsseldorf, Germany; ^2^ Department of Neurology, Maria Hilf Clinic, Moenchengladbach, Germany; ^3^ Department of Neurology, Palacky University in Olomouc, Olomouc, Czechia; ^4^ Department of Neurology, Barzilai Medical Center, Ashkelon, Israel; ^5^ Brain and Mind Center, Medical Faculty, The University of Sydney, Sydney, NSW, Australia

**Keywords:** B cell depletion, multiple sclerosis (MS), neuromyelitisoptica spectrum disorders (NMOSD), myelin oligodendrocyte glycoprotein associated autoimmune disease (MOGAD), autoimmune disease of the central nervous system

## Abstract

Cumulative evidence along several lines indicates that B cells play an important role in the pathological course of multiple sclerosis (MS), neuromyelitisoptica spectrum disorders (NMOSD) and related CNS diseases. This has prompted extensive research in exploring the utility of targeting B cells to contain disease activity in these disorders. In this review, we first recapitulate the development of B cells from their origin in the bone marrow to their migration to the periphery, including the expression of therapy-relevant surface immunoglobulin isotypes. Not only the ability of B cells to produce cytokines and immunoglobulins seems to be essential in driving neuroinflammation, but also their regulatory functions strongly impact pathobiology. We then critically assess studies of B cell depleting therapies, including CD20 and CD19 targeting monoclonal antibodies, as well as the new class of B cell modulating substances, Bruton´s tyrosinekinase (BTK) inhibitors, in MS, NMOSD and MOGAD.

## 1 Introduction

The fundamental role of B cells in the pathogenesis of inflammatory central nervous system (CNS) disease has emerged through extensive studies in the last 10-15 years. However, the exact role of B cells in the development of these disorders and the mechanisms of the drugs targeting B lymphocytes still remain, at least in parts, unclear.

Multiple Sclerosis (MS) is an inflammatory, autoimmune disorder of the CNS characterized by demyelination and axonal loss. While demyelination, in principle, is reversible, neuroaxonal degeneration is almost invariably permanent. Therefore, new therapies are urgently needed to effectively prevent chronic neurodegeneration as the main determination of long term disability. Immunpathological changes in MS are generally characterized by activity and complex interactions of T-cells, myeloid-cells, and B cells (1, 2).

For a long time, neuromyelitisoptica (NMO), was considered to be a rare, special variant of MS. However, in 2004, a specific antibody neuromyelitisoptica immunoglobulin G (NMO-IgG) (3), one year later identified to be directed against the water channel aquaporin-4 (AQP-4) was discovered (4). This established NMO as a distinct entity in its own right and later allowed to broaden its clinical manifestation for which the term neuromyelitisoptica spectrum disorders was coined. These observations fundamentally changed diagnostics and treatment of this group of inflammatory CNS disorders. Further research revealed elevated B cell and plasmablast activity and attenuated B cell regulatory function and complement-mediated astrocyte damage (5) underlying the pathobiology of NMOSD (6). In addition, the detection of a specific antibody in NMOSD stimulated efforts to look for specific markers and subtypes in MS that continue to this day (7).

Similar to MS, myelin oligodendrocyte glycoprotein (MOG)-antibody-associated autoimmune disease (MOGAD) is an inflammatory, demyelinating disease of the CNS with reference to oligodendrocytes, which is primarily characterized by (mostly relapsing) optic neuritis, myelitis and brainstem encephalitis or acute disseminated encephalomyelitis (ADEM) in children (8–11). In contrast to MS and MOGAD, NMOSD particularly affects astrocytes. On the other hand, NMOSD and MOGAD, with their predilection sites in the optic nerves, cerebellum, brain stem and spinal cord (causing long spinal cord lesions), have more in common with each other than with MS (10).In terms of specific MRI features, MOGAD is characterized by anterior participation of the optic nerve involving the peribulbar fat, poorly delineated (‘fluffy’) lesions and central grey matter of the spinal cord (in axial imaging - ‘H-sign’). However, in MOGAD, MRI lesions may regress and a positive MOG-Ab status may transform to seronegativity (11). Neuropathologically, CD4+ T-cells dominate MOGAD lesions, whereas MS is dominated by CD8+ cells. To date, there is no female predominance in MOGAD, which distinguishes the disease from MS and NMOSD (11). Overall, MOGAD is an independent clinical entity that sits between MS and NMOSD in the spectrum of autoimmune inflammatory diseases. It represents around 40% of the patients presenting as NMOSD patients who are AQP4 antibody negative (9).

In the following review, we provide a brief recapitulation of B cell development, their involvement in the pathology of MS and other inflammatory demyelinating CNS disease and provide a detailed overview of B cell depleting therapies and key clinical studies.

## 2 B cell development – from bone marrow to periphery

Myeloid and erythroid progenitor cells as well as lymphoid progenitor cells differentiate from self-renewing pluripotent haematopoietic stem cells of the bone marrow, whereas from the latter, mature B cells develop through various intermediate stages. Up to the stage of “immature B cell”, rearrangement of the immunoglobulin segment genes occurs, resulting in the expression of a mature B cell receptor (BCR) that consists of two heavy and two light chains (12–14).

**Figure 1:**
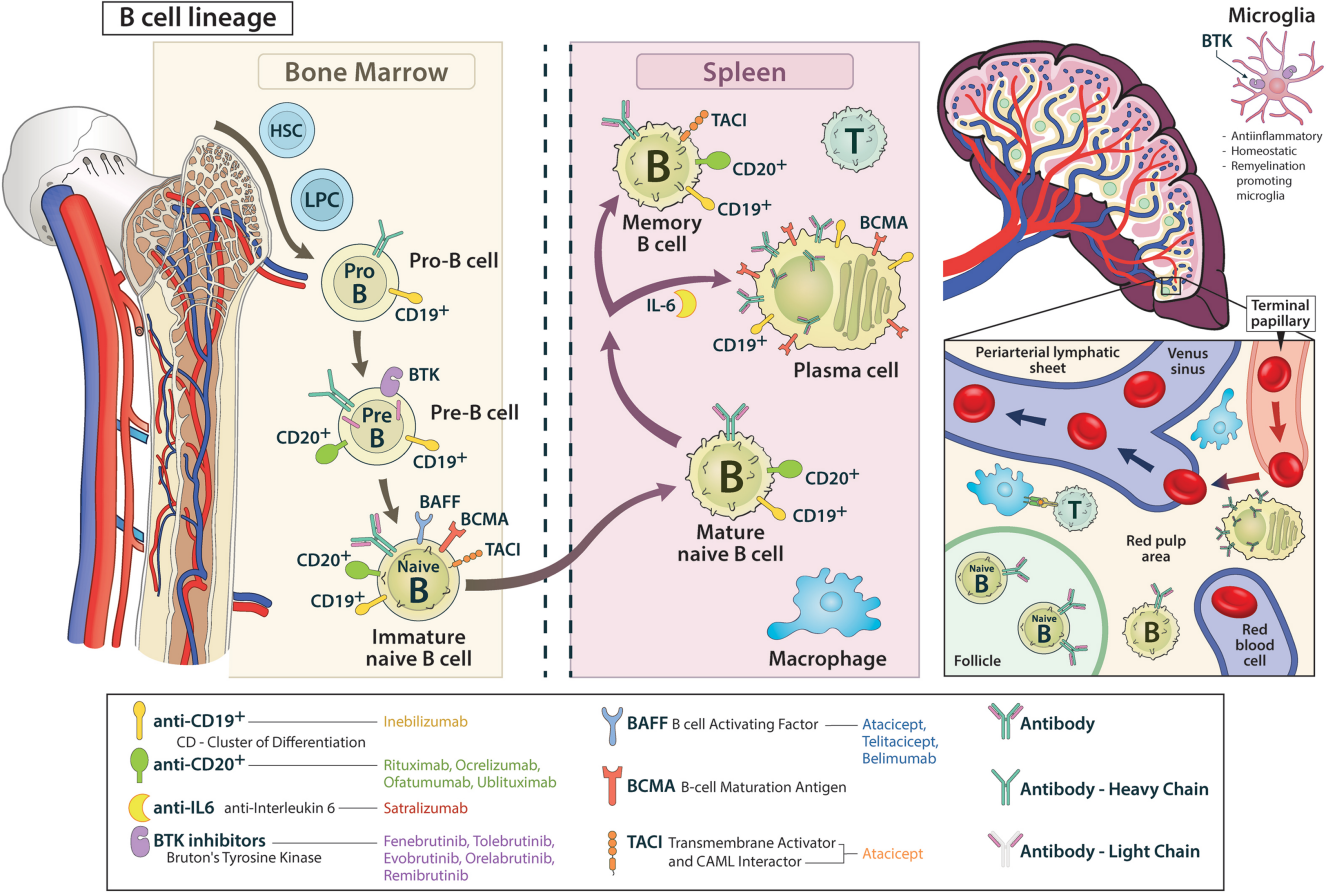
The B cell lineage from bone marrow to the periphery is shown, including relevant surface proteins and their targeting by corresponding drugs.

In the pro-B cell stage, the gene segments of the heavy chain are rearranged and ultimately expressed as a µ-chain. While the µ-chain represents the heavy chain of the final BCR product, the CD19 antigen is already expressed on the pro-B cell (15). In the pre-B cell stage, CD20 is already displayed on the cell surface and the light chain gene segments of the BCR are reorganized, however not yet integrated into the receptor. Among other actions the signaling pathway of Bruton’s tyrosine kinase (BTK) stimulates the development of the immature B cell, in which light and heavy chains are combined to form the BCR on the cell surface (16–19). The immature B cells then undergo two selection processes: First, they interact with endogenous antigens (AG) of the bone marrow. Afterwards, the immature B cells migrate from the bone marrow and are challenged with exogenous AGs in the spleen and secondary lymphatic organs by contact with macrophages, amongst others. In the course of this process, signaling with B cell activating factor receptor (BAFF-R), B cell maturation antigen receptors (BCMA-R) and transmembrane activator and CAML interactor receptors (TACI-R) leads to the development of an immature B cell to a mature-naïve B cell. By interaction with follicular T helper cells, differentiation into memory B cells and short-lived AG-producing plasmablasts is initiated. In this process, BAFF-R, BCMA-R and TACI-R are also heavily involved. While TACI-R is mainly expressed on memory cells, BCMA-R is found on plamablasts/plasma cells, BAFF-R is expressed on cells from the immature B cell onwards (20–22). Memory cells express CD19 and CD20, whereas only CD19 is found on plasmablast (12, 13, 15, 17, 19, 23–26) (**Figure 1**).

## 3 Complex immunological role of B cells in MS, NMOSD and MOGAD

The presence of oligoclonal bands in the cerebrospinal fluid, detection of circulating CNS-reactive antibodies and demonstration of immunoglobulins deposited in CNS lesions of MS patients directed for long the focus of interest primarily on the antibody-producing function of B cells. The impressive and rapid improvement observed after drug-induced depletion of B cells in MS prompted reconsideration of the key role of B lymphocytes in the immunopathogenesis of MS. Along with other evidence, these effects suggested a role of B cells as antigen presenting cells that would initiate or amplify T-cell responses to CNS antigens and emphasized their roles as complex players diversely interacting with and modulating several other components of the immune system (16, 19, 23, 25, 27–32) and which can now be traced even at the epigenetic level (33).

The pathobiology of MS is assumed to involve interactions of multiple intrinsic (e.g. genetic variants) and extrinsic (e.g. Epstein-Barr-virus - EBV - infections, vitamin D deficiency) factors. This leads to an autoreactive activation of peripheral immune cells such as CD8+ T-cells and CD4+ T-cells (e. g. TH-17 cells as a subset of CD4+ T-cells) paralleled by a breakdown of immune-tolerance. These cells migrate *via* meningeal vessels or choroid plexus structures across the blood-CSF barrier into the brain, get reactivated by local CNS autoantigens presented by resident glial and other myeloid cells and attack endogenous CNS structures (1, 34–39). The activation process of autoreactive T-cells is flanked by a deficiency of immune-inhibitory components, with both a quantitative deficiency of T-reg cells and their dysfunction due to a newly developed resistance of autoreactive T-cells (34, 37). Beyond the blood-brain barrier, innate microglia also play an important role in the progression of MS, although this role is not yet fully understood. On the one hand, microglial cells can act in a regenerative manner by promoting neuron recovery and remyelination *via* phagocytosis of cell debris or modulation of synaptic connections. In addition these cells can also exert CNS-destructive actions *via* the regulation of cytokine and chemokine release (40). This leads to an additional recruitment of macrophages and lymphocytes, which amplifies the already existing autoimmune inflammatory processes in the CNS (35, 41–45).

**Figure 2:**
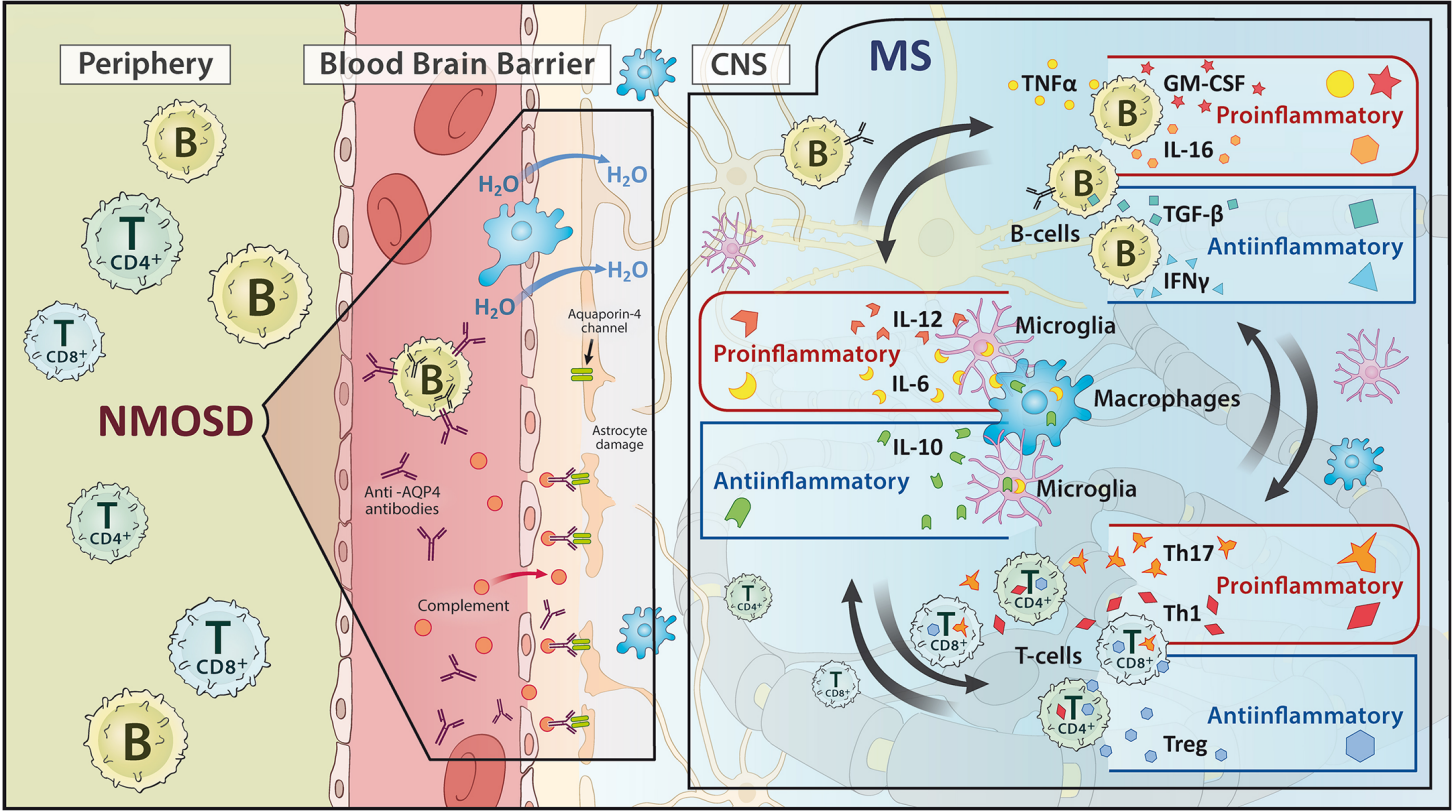
Schematic representation of the pathomechanism of (left) neuromyelitis optica spectrum disorders (NMOSD) and (right) multiple sclerosis (MS). While NMOSD focuses on an antibody reaction, MS shows an interplay of pro- and anti-inflammatory processes between B cells, T cells and microglia/macrophages.

B cells are also essentially involved in the outlined process. Through internalization and consecutive antigen presentation *via* the major histocompatibility complex II (MHC II), B cells stimulate and activate T-cells. In addition, they secrete pro- (e.g. TNF-α, GM-CSF) and anti-inflammatory (e.g. IL-10, IL-35) cytokines that modulate the invasion and behavior of both, the innate immune system in the form of macrophages or microglia and the adaptive immune system such as T-cells (19, 23, 25, 27–31). Recently, the importance of the dura with its lymphatic vessels has been recognized as a particularly relevant site for the residency and development of B cells, enabling communication across the blood-brain barrier (23, 32, 46, 47). Late-stage tertiary follicles of B cells in the meninges of MS patients have been demonstrated to maintain and modulate continuous inflammation (15, 19, 48, 49). Complementing this, the gut-brain axis is also coming into focus as an important component of the B-cell modulated autoimmune processes underlying MS. The determination of IgA-bound specific intestinal taxa in MS patients and experiments in animal models indicate an immunomodulatory function of IgA-producing B cells that receive their imprinting in the intestine and migrate to the CNS (50, 51). Unaffected by this, the ability of B cells to produce antibodies seems to play a subordinate role. The IgG antibodies detected in the CSF are rather to be regarded as a reaction to ubiquitous intracellular proteins that are produced as part of the CNS destruction process occurring during the disease (28, 30) (**Figure 2**, right).

Nevertheless, recent experiments have demonstrated that monoclonal-Ab of B cells from CSF and serum of MS patients bind the EBV transcription factor EBNA1 with high affinity. They also show molecular mimicry with CNS cell adhesion molecule GlialCAM. This observation leads to the hypothesis that after contact with EBV, B cells enter the CNS/CSF space, encounter GlialCAM antigen and then undergo affinity maturation, producing high-affinity clones for anti-GlialCAM-Ab (52). This may confirm the long-established link between MS and EBV infection, which has recently been established in a cohort of 10 million young adults (53).

The situation is different, however, in NMOSD. In 70-80% of all NMOSD patients, aquaporin-4 immunoglobulin G antibodies (AQP4-Ab) can be detected, which are directed against the body’s endogenous AQP4 antigen. Up to 42% of AQP4 IgG negative patients harbor IgG antibodies to Myelin Oligodendrocyte Glycoprotein (MOG) (54). There may be overlap with or genuine MOG antibody associated disease (MOGAD) (55). AQP4 is found mainly in the central nervous system on astrocytes near the blood-brain barrier and function as a water channel, whereas the binding of AP4-Ab to AQP4 channels causes their downregulation. This leads to intra-myelinoedema due to disturbed water homeostasis and to an activation of the complement system with assembly of the terminal complement complex C5b-9 and consecutive necrosis of the cells expressing AQP4. Leukocytes migrate through the pre-damaged blood-brain barrier, further promoting the pathological process (15, 25) (**Figure 2**, left). Interestingly, there is also information on the possible involvement of molecular mimicry in the pathogenesis of NMOSD. Reports have appeared on the autoimmune cross-reactivity of T cells with the protein adenosine triphosphate-binding cassette of the intestinal bacterium Clostridium perfringens (56). Evidence how this impinges B cell responses has not yet been put forth.

## 4 Targets of B cell therapies

Several B cell targeting strategies have been approved in recent years, some of which are the most effective in treating MS and other inflammatory CNS disease. In the following, the B cell depleting therapies are listed, with the key studies, also summarized in **Supplementary Table 1**.

### 4.1 CD20 antigen related drugs

**Figure 3:**
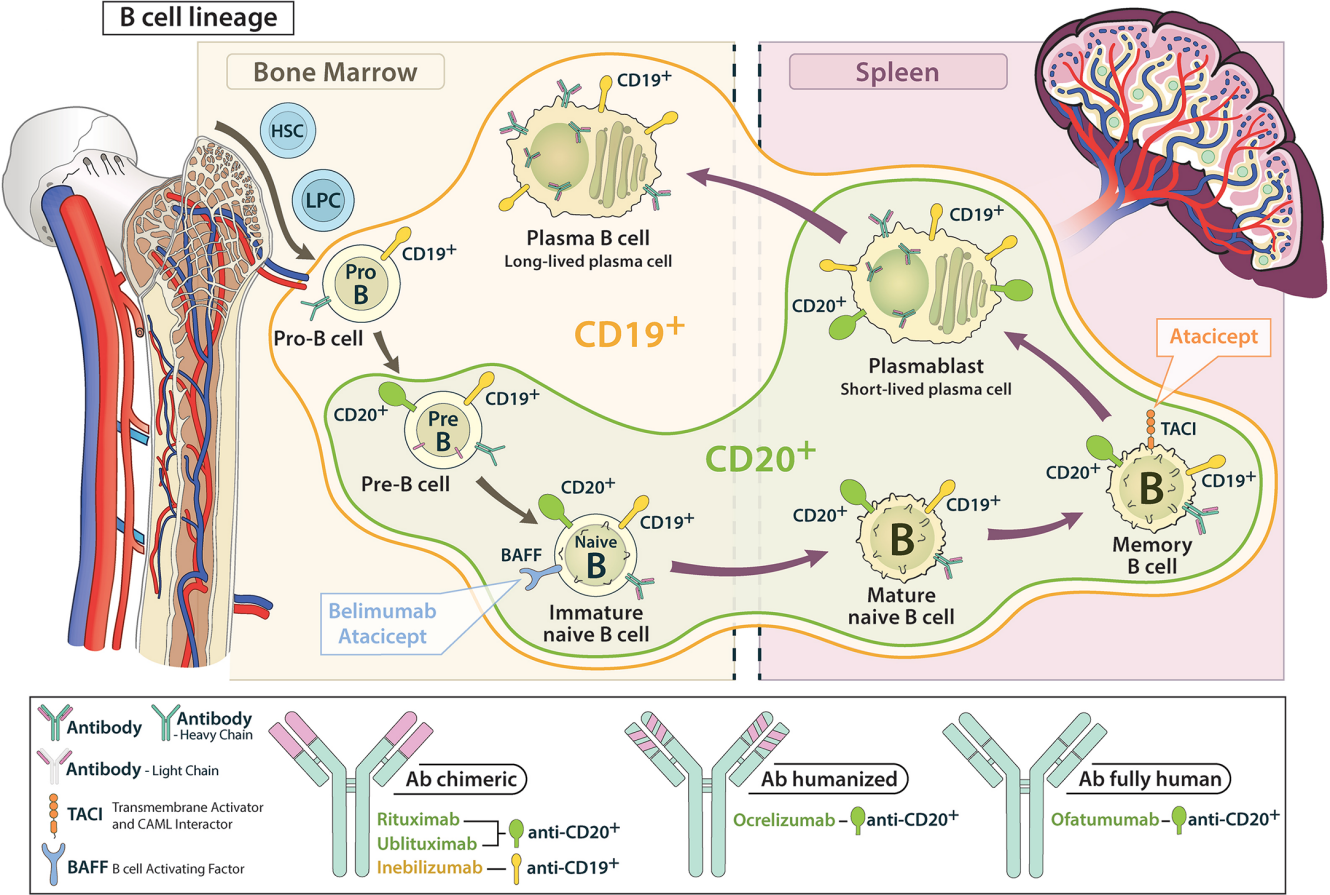
Schematic representation of the attack range of CD 19 and CD 20 antibodies in the B cell lineage. CD 19 antibodies cover earlier and later developmental stages compared to CD 20 antibodies.

CD20 is a four-transmembrane protein expressed on the surface of pre-B cells up to memory cells, but not on the long-lived and antibody-producing plasma cells or stem cells and pro-B cells. Thus, targeting CD20 does not interfere directly with antibody production or generation of new B cells in the bone marrow (**Figure 3**).

#### 4.1.1 Rituximab

Rituximab was the first CD20-targeting antibody to be tested in people with MS. As a chimeric monoclonal antibody (Ab), it depletes B cells primarily *via* complement-dependent cytotoxicity (CDC) (28–30, 57).

In 2008, a phase 1 study confirmed the safety of two cycles of rituximab in 26 relapsing-remitting multiple sclerosis (RMS) patients. The results for the secondary endpoint also showed a decrease in gadolinium-enhancing T1 lesions and new T2 lesions down to zero at 72 weeks (58). In the double-blind phase 2 HERMES trail, superiority of rituximab over placebo was demonstrated for the primary endpoint namely reduction in the total number of gadolinium-enhancing lesions on MRI at 12, 16, 20 and 24 weeks. In addition, an improvement in clinical outcomes such as the reduction in relapse rate within the first 24 weeks could be achieved as secondary endpoints (59).

The positive outcome of the RMS studies could not be replicated in studies of patients with primary progressive multiple sclerosis (PPMS). In the double-blind phase 2/3 OLYMPUS trial, there was no significant difference between placebo and rituximab treated patients in the primary endpoint of time to confirmed disease progression. However, in a subgroup analysis, rituximab attenuated disease progression in PPMS patients younger than 51 years of age with gadolinium-enhancing lesions on MRI compared to placebo (60).

In an initial open-label pilot trial in patients with NMO in 2005 rituximab demonstrated beneficial effects in 8 patients (61). Subsequently, a number of retro- and prospective studies followed. However, no randomized double-blind studies were conducted (62, 63) until 2020 when the Japanese RIN-1 study with rituximab in 38 patients with NMOSD was published. The primary endpoint in this study, time to first relapse within 72 weeks, was reached. While no patient relapsed on rituximab, 7 patients suffered attacks on placebo (62, 63). Due to the limitation of the small number of patients and the lack of an independent relapse assessment as well as the exclusive inclusion of aquaporin-4-antibody positive patients the study leaves several questions unanswered (62, 63).

A similar conclusion regarding the efficacy of rituximabin myelin-oligodendrocyte-glycoprotein-immunoglobulinG-associated disorders (MOGAD) can be drawn. After numerous smaller studies (64), a prospective study comprising 102 paediatric MOGAD patients from 8 countries compared the different treatment protocols of the respective centers along with their outucomes between 2014 and 2016. Treatment of 9 children with rituximab resulted in a decrease in the annualized relapse rate from 2.12 to 0.67, with the median Expanded Disability Status Scale (EDSS) score remaining stable (65). In 2020, a retrospective study of 121 pediatric and adult patients from 13 countries found rituximab to be effective. In the group of patients who had relapsed before switching to rituximab, 70.0% remained relapse-free for the next 11.2 months, and in the group of patients with two or more relapses, 52.5% did not experience an attack within the subsequent 12.1 months (66). In 2022, a first systematic meta-analysis of 13 studies and a total of 238 patients was published. In the analyzed data, 55% of the patients treated with rituximab did not have a relapse within the observed study periods (67). The following rituximab-specific adverse reactions have manifested in the pivotal trials and in clinical use: Infusion-associated reactions such as fever, tachycardia and hypotension, upper respiratory tract infections and changes in the blood count in a sense of neutro- or thrombocytopenia (16, 23, 28, 67). In summary, the current data principally supports the efficacy of rituximab in MOGAD, however, the exact mode of action in this disease still remains unclear, as relapses occurred despite a significantly reduced number of B lymphocytes (41, 43).

Regardless of the above, there is currently a lack of randomized controlled trials on therapeutic options for MOGAD. A 152-patient MOGAD trial is currently recruiting to test the efficacy and safety profile of the interleukin-6 antagonist sartralizumab against placebo (ClinicalTrials.gov Identifier: NCT05271409). Currently, treatment is guided according to expert opinion and case series: Acute MOGAD attacks in adults are treated with an i.v. steroid course followed by a 2-3 months tapering. Depending on the clinical situation, a maintenance therapy with rituximab, azathioprine, mycophenolatemofetil or IVIG follows. This does not apply to children, who often have a monophasic course. Both the phasing out of cortisone with regard to possible side effects and the initiation of a basic therapy must be critically discussed (10, 68, 69).

#### 4.1.2 Ocrelizumab

Ocrelizumab was developed based on the experience with rituximab and was the first CD20-based receive approval for MS. This humanized monoclonal antibody (Ab) depletes B cells predominantly *via* antibody-dependent cellular cytotoxicity (ADCC) (29, 30, 70).

In 2011, a double-blind randomized phase 2 trial tested ocrelizumab at doses of 600 and 2000 mg for efficacy compared to placebo or intramuscular interferon β (IFNβ)-1a in 220 study participants with RMS for a total of 48 weeks. The number of gadolinium-positive T1 lesions at weeks 12, 16, 20 and 24 (the primary endpoint) were significantly lower in the two ocrelizumab groups compared to the placebo and comparator groups. A significant difference in efficacy between the two doses of ocrelizumab could not be determined (71). This trial added support to the role of B cells in MS pathogenesis and prompted the double-blind, randomized, double-blind OPERA-I and II phase 3 trials in 1656 relapsing MS patients, which led to the approval of ocrelizumab by the regulatory authorities FDA, European Medicines Agency (EMA), Health Canada and Therapeutic Goods Administration for the treatment of relapsing MS. After 96 weeks, the primary endpoint of a lower annualized relapse rate compared to IFNβ-1a was achieved. Ocrelizumab also proved to be superior to IFN-β in most secondary clinical and MRI endpoints. However, superiority in the items of improvement in multiple sclerosis function score, including Short Form Health Survey-36 (SF-36 score), and total brain volume loss were not significant. In addition, a higher number of infusion reactions and twice the absolute number of neoplasia manifestations (4 vs. 2) occurred with ocrelizumab compared to interferon-β. Consequently, the study addressed the need for further long-term follow-up observations of the patients treated with ocrelizumab (72). In the open-label extension patients treated initially with IFNβ were switched to ocrelizumab. After 3 years, the population treated with ocrelizumab from the beginning had a lower percentage of disease progression over 24 weeks and a reduced loss of brain volume (-1.87% versus -2.15%). Importantly, the safety profile, now collected over the course of 5 years, was similar to the core study (73).

Using the evidence from the OLYMPUS study the double-blind, randomized ORATORIO 2017 trial investigated the effect of ocrelizumab compared to placebo in 732 patients with PPMS (27). The percentage of patients on ocrelizumab who experienced disability progression at 12 weeks (primary endpoint) was 32.9% compared to 39.3% in the placebo group (24% reduction, p=0.03). In addition, ocrelizumab met all secondary clinical and MRI endpoints except for an improvement in the SF-36. Compared to the placebo group, more infusion reactions up to the intermediate category and an increased incidence of neoplasia were observed with ocrelizumab (74). It should be noted that ORATORIO primarily included PPMS patients with short disease duration (16). However, recent *post-hoc* data suggest efficacy of ocrelizumab even in the presence of increased disability due to MS (75). In an open-label ORATORIO follow-up study, a significant improvement in 24-week confirmed disability progression was still demonstrated with ocrelizumab for up to 6.5 years after the start of the study compared to patients who switched from placebo to ocrelizumab after the official end of ORATORIO (33.3% versus 44.7%). There were no new safety findings (76).

Direct comparisons of ocrelizumab with other disease-modifying therapies (DMT) in a double-blind and randomized study design are still lacking, with the exception of IFNβ. A comparative study between rituximab and ocrelizumab is recruiting (ClinicalTrials.gov Identifier: NCT04578639). Currently, a systematic meta-analysis from 2019 provides the most robust data. It confirms, that ocrelizumab has an efficacy and safety profile that is superior or comparable to other available DMT at all endpoints, with the exception of natalizumab and alemtuzumab (77). The efficacy of switching to ocrelizumab after treatment failure with another DMT has been explored in two prospective non-randomized studies. The North American CHORDS study includes a population of 576 patients with RMS with a clinically or radiologically objectified relapse on one or more DMTs for at least 6 months. The primary endpoint “No evidence of disease activity” (NEDA) includes the absence of protocol-defined relapses, confirmed disability progression, T1 Gd-enlarging lesions and new/enlarging T2 lesions. At 96 months, 48.1% of patients reached this point on ocrelizumab (78). The European counterpart study CASTING, which included 680 patients with the same baseline characteristics, 74.8% achieved NEDA after 96 weeks (79). Independently of this, a non-blinded open-label study on the safety and efficacy of the use of ocrelizumab in particularly early stages of RMS is currently ongoing (EudraCT Number: 2016-002937-31).In 2020, a sub-study of CASTING, called ENSEMBLE PLUS, showed that shortening the infusion time for the second administration of ocrelizumab from 3.5 to 2 hours was associated with a similar percentage of infusion-related adverse events (23.1% in the 3.5-hour group and 24.6% in the 2-hour group) (80).

In the pivotal studies and clinical use, the following ocrelizumab-specific adverse reactions have been noted: Infusion-associated reactions such as fever, tachycardia and hypotension, upper respiratory tract infections, increased herpes and influenza infection rates and opportunistic infections including PML (81, 82).

#### 4.1.3 Ofatumumab

Ofatumumab is a fully humanCD-20-monoclonal antibody that, unlike ocrelizumab, is administered subcutaneously at monthly intervals. It acts primarily through CDC and was approved by the FDA in August 2020 and by EMA in March 2021 for the treatment of MS (16, 19, 28–30, 58, 83).

The MIRROR phase 2 study demonstrated efficacy of ofatumumab at doses of 3, 30 or 60 mg every 12 weeks or 60 mg every 4 weeks compared to placebo in the primary endpoint, the cumulative number of new gadolinium-enhancing T1 lesions at weeks 4, 8, and 12. The cumulative number of new lesions was reduced by 65% in all ofatumumab dose groups compared to placebo at 12 weeks. Interestingly, all ofatumumab doses achieved radiological efficacy, and the authors concluded that complete depletion of CD19-B cells was not necessary for a robust effect (84). The twin studies ASCLEPIOS I/II, a randomized-blinded and comparator-controlled phase 3 study with ofatumumab in patients with RMS was published in 2020. In this 30-month period, ofatumumab versus teriflunomide met its primary endpoint, the adjusted annualized relapse rate (0.11/0.1 for ofatumumab versus 0.22/0.25 for teriflunomide). Among neurofilament light chains (Nfl) in the serum of ofatumumab patients was reduced compared to teriflunomide at 3, 12 and 24 months. However, this sign of a neuroprotective effect of ofatumumab contrasts with the lack of difference between the two drug groups in terms of brain volume change over time (85). The phase 3b ALITHOS study, designed as an open-label, long-term study, collected cumulative data from 1969 patients from the previously mentioned studies. It included patients who continued treatment with ofatumumab after ASCLEPIOSI/II and the phase 2 trials or who were switched from teriflunomide to ofatumumab after the end of ASCLEPIOS. In the cumulative patient data with ofatumumab exposure of up to 3.5 years, there were no new safety signals compared to those in the ASCLEPIOSI/II trial (83).

The SOSTOS trial (ClinicalTrials.gov Identifier: NCT05090371) is currently recruiting patients who switch from platform therapies to after a proven increase in Nfl concentration. Under the name ARTIOS, the switch from dimethyl fumarate or fingolimod to ofatumumab due to persistent disease activity is being investigated in a planned trial of 555 people (ClinicalTrials.gov Identifier: NCT04353492). In addition, the OKLIOS trial is recruiting patients to evaluate the impact of switching from another CD20-mAB (most likely ocrelizumab) to ofatumumab in terms of safety and efficacy (ClinicalTrials.gov Identifier: NCT04486716). Results are currently pending.

According to studies and clinical use, the following adverse reactions should be considered when using ofatumumab: an increased rate of infection, especially of the upper respiratory tract and in pneumonia and urinary tract infections, systemic and local reaction to the administering injection, a lowered IgM level and hepatitis B reactivation (81, 83).

#### 4.1.4 Ublituximab

Ublituximab is a chimeric glycoengineered IgG1 monoclonal antibody that has been modified at the constant fragment (Fc) region and acts mainly *via* ADCC on CD20-positive cells. It has been argued that the higher binding affinity will result in dose reduction and faster infusion times compared to previous anti-CD20 therapies (16, 30, 86, 87). In December 2022, the drug was approved by the FDA for the treatment of patients with RMS (88).

In a phase 2 study published in 2021 (n = 48 RMS patients), total doses over 24 weeks of 1050 to 1350 mg with infusion times of 1 to 4 hours were applied and each dose/infusion time group was controlled against a placebo group. The primary endpoint, the proportion of patients treated with ublituximab with ≧̸95% peripheral CD19+ B-cell depletion at 2 weeks after ublituximab application, was met at 100% by all dose/infusion time groups. Furthermore, an increase in infusion-related systematic reactions was not found to be related to higher doses or shorter infusion times (87). The results of 2 phase 3 randomized, double-blind twin trials (ULTIMATEI/II) were published in 2022. 1094 RMS patients were treated with either ublituximab or teriflunomide for 96 weeks according to a standardized protocol. The primary endpoint, annualized relapse rate over 96 weeks, was met by ublituximab at 0.08 and 0.09 (ULTIMATEI/II) versus teriflunomide at 0.19 and 0.18 respectively. In the secondary endpoints, although ublituximab resulted in fewer MRI brain lesions compared to teriflunomide with effective CD19 depletion, significant differences in disability worsening could not be substantiated, probably due to the low rate of disability progression in both groups (86).

Looking at applications in NMOSD, Mealy et al. presented a phase 1 study in 2019 in which active NMOSD patients were administered a single dose of ublituximab in addition to 1 g of methylprednisolone for 5 days. In the absence of serious adverse events within 90 days, the primary endpoint of safety of additional ublituximab use was met. The secondary endpoints showed a decrease in the median EDSS score from 6.5 to 4.0 within 90 days. However, with a group size of only 5 AQP4-IgG-seropositive NMOSD patients and the phase 1 study design, the significance of the study is limited (89).

Based on the data to date, the safety profile of ublituximab is similar to that of the other CD20 antibodies: leading adverse events are infusion-related reactions with fever and tachycardia, upper respiratory tract infections and headache (86). An open-label observational study is currently being conducted following ULTIMATEI/II to better explore the long-term safety profile of ublituximab (ClinicalTrials.gov Identifier: NCT04130997).

#### 4.1.5 Covid 19 and CD20 depleting basic therapies

The question of what the pandemic means for MS patients on immunomodulatory therapy arose early in Covid-19. Retrospective data indicate that factors such as female gender, comorbidities, previous drug escalation of DMT and hospitalization predispose MS patients to infection with covid-19 (90). In particular, the question is about the influence of DMT on the efficacy of vaccination. It is known from studies that various aspects such as vaccination timing and type of vaccine differ between the different basic therapy classes. In particular, a reduced humoral response after vaccination has already been described for CD20-depleting drugs and sphingosine-1-phosphate receptor modulators (91). This has also been confirmed in numerous prospective, retrospective and experimental studies: Under CD20-depleting and sphingosine-phosphate-modulating therapies, there is a reduced humoral response compared to vaccine naïve subjects compared to the other MS medications (91–99). At the same time, there is evidence that the reduced humoral response is accompanied by an increased T-cell response (95, 99). Whether the increased T-cell response can compensate for the decreased humoral response in relation to covid infection remains unclear and is currently the subject of research (92, 98). With regard to patients on anti-CD20 therapy, the current literature contains conflicting statements on whether CD20-depleting therapy leads to an increased likelihood of hospitalization (100, 101). Remarkable are new findings on different vaccination regimens that have direct therapeutic consequences. It could be shown that, especially with CD20-depleting therapies, different vaccines have more or less strong effects on the long-term humoral response after vaccination. The choice of a specific vaccine is therefore of particular importance (97). On the other hand, after a third Covid booster vaccination, a significant increase in both the humoral - and the T-cell response is observed (94).

### 4.2 CD19 antigen directed drugs

CD19 is a type I transmembrane glycoprotein that, in contrast to CD20, is already expressed on both early pro-B cells and late antibody-producing plasmablasts and some plasma cells during B-cell development. Accordingly, CD19 is thought to be more important in mainly antibody-driven diseases such as NMOSD (15, 25, 64, 102, 103).

#### 4.2.1 Inebilizumab

Inebilizumab is a CD-19 directed humanized monoclonal antibody that has an increased affinity to the Fc region due to its glycosylation and it eliminates CD-19 positive B cells primarily *via* ADCC. It was approved by the FDA in 2020 for the treatment of seropositive NMOSD patients, in 2021 in Japan for the prevention of clinical relapses in NMOSD and by EMA as monotherapy for the treatment of adult patient with seropostive NMOSD in 2022 (15, 25, 64, 102, 103).

In 2017, a phase 1 study assessed the safety and tolerability of intravenous or subcutaneous inebilizumab (at 30, 100, 600 mg iv - 60, 300 mg sc) compared to a corresponding placebo group. With good tolerability, the most common side effects were nasopharyngitis, upper respiratory tract infection, urinary tract infection and infusion/injection-related side effects. With decreasing immunoglobulin levels, there was no change in pre-existing anti tetanus toxoid IgG levels (103). The randomized multicenter phase 2/3 N-MOmentum trial (2019) tested the efficacy of inebilizumab versus placebo in a population of 230 mostly seropositive but also seronegative NMOSD patients. The primary endpoint of the study was the number of days to clinical relapse within the observed time point. Within the observation period, 12% of the inebilizumab group relapsed compared to 39% of the placebo group. There were no new safety profile findings, although one death occurred in aninebilizumab-treated patient after initial administration, with two new lesions on MRI involving both white and grey matter. The presence of progressive multifocal leukencephalopathy could neither be excluded nor confirmed with certainty. Irrespective of this, the antitetanus toxoid IgG levels were also stable in N-MOmentum, therefore it can be assumed that there is no influence on the vaccination response achieved before the initial administration of inebilizumab (104). As rituximab alone was approved for the treatment of NMOSD prior to the FDA approval of inebilizumab, it is relevant to ask whether inebilizumab could be considered as an alternative drug for NMOSD patients who had relapsed on rituximab. In a *post-hoc* analysis of the N-MOmentum data from 2022 including 17 patients who had initially taken rituximab, the efficacy of inebilizumab as a second therapy was confirmed. However, this patient population showed an increased susceptibility to infections (102).

The previous findings on side effects of inebilizumab were based on 4 years of drug use, which emerged in a *post hoc* analysis on data from the N-MOmentum study and its extension (26): As known from anti-CD20-Ab’s, infusion-related reactions with fever and tachycardia, upper respiratory tract infections as well as urinary tract infections are worth mentioning. However, there was no case of progressive multifocal leukencephalopathy.

### 4.3 Cytokine antagonists

BAFF (B-Cell Activating Factor) and APRIL (A proliferation inducing ligand), members of the TNF supra family, are important regulatory cytokines in B cell development and activation. They exert their effect upon interaction with receptors of the BAFF, TACI and BCMA signaling pathways (19, 22, 30). Attempts have been made to interfere with these pathways in the treatment of MS with the drugs atacicept and belimumab. Interleukin 6 represents an important component in the pathogenesis of NMOSD, addressing B cells among others (105). Sartralizumab is an approved drug for the treatment of NMOSD.

#### 4.3.1 Atacicept

Atacicept is a recombinant fusion protein of the extracellular TACI domain and human Fc IgG moiety that binds to the cytokines BAFF and APRIL, preventing their interaction with the B-cell surface receptors (8, 19, 41, 72). It acts selectively on B-cell development by blocking plasma cells and late B-cell development, but has no effect on B-cell progenitors or memory cells (106).

In the ATAMS phase 2 study, 255 RMS patients were randomized 1:1:1:1 to three dose groups of atacicept (25 mg, 75 mg, 150 mg) and a placebo group. The primary endpoint was the change in the mean number of gadolinium-enhancing lesions on T1-weighted MRI per patient. However, there was early study discontinuation due to increased annualized relapse rates in all atacicept groups compared to placebo (atacicept 25 mg 0.86, 75 mg 0.79, 150 mg 0.98 versus placebo 0.38). Also, no significant group differences were found in the number of gadolinium-enhancing T1 lesions (107). After these negative results emerged, the phase 2 ATON trial, which was conducted at the same time, was reviewed. This was a randomized clinical trial that tested atacicept against placebo in 34 patients with unilateral optic neuritis without a definitive MS diagnosis. The review of the study results led to early discontinuation by the sponsor. Although there was evidence of reduced axonal retinal layer thickness loss in the atacicept group (-8.6 µm versus -17.3 µm in the placebo group), there was a concurrent significantly increased conversion rate to definitive MS in the same group (35.3% versus 17.6%) (46).

Numerous hypotheses have been proposed regarding the reasons for MS worsening with atacicept. It is now generally agreed that atacicept, through its binding of APRIL, shifts the immunological balance of inhibition and excitation of the immune system to the disadvantage of inhibition (19, 22, 30, 108). APRIL has a higher affinity for TACI and BMCA-R and is, if this interaction is disturbed, an increased interaction of T and B cells and consequently, increased MS activity, occurs. Insights to confirm this presumed mechanism could be gained by considering pure BAFF antagonists such as tabalumab and belimumab, which do not compromise APRIL interactions. For example, belimumab was approved for the treatment of systemic lupus due to its efficacy, but studies of its use in MS showed neither improvement nor worsening (22). In addition, unlike CD20 monoclonal antibodies, atacicept does not deplete memory B-cells, which are also known to modulate T-cells. Consequently, these elective interference with the immunological balance is again discussed as a reason for worsening of the MS progression with atacicept (20, 22, 30, 108). Other possible explanations for the failure of Atacicept include the reduction in plasma cells that secrete the regulatory cytokine IL 35 (109).

#### 4.3.2 Telitacicept

Telitacicept, as an analogue of atacicept, is also a recombinant fusion protein of the extracellular TACI domain and the humanized Fc-IgG part, which binds to the cytokines BAFF and APRIL and thus prevents their interactions with B-cells (110). Unlike atacicept, it has a longer TACI fragment (111).

In an open-label, uncontrolled phase 2 trial in China, 8 patients with relapsing NMOSD were treated with telitacicept once weekly for an additional 46 weeks following three cycles of plasma separation. The primary endpoint was the time to first relapse during 48 weeks of observation. Two patients (25%) relapsed and five patients (63%) remained relapse-free after 48 weeks of treatment. The relapse of the two patients occurred after a longer inter-relapse interval than prior to study inclusion (112). Thus, this cytokine antagonist could be a valuable treatment for NMOSD, probably due to the different underlying pathology of MS and NMOSD and its effect on antibody production. A phase 3 trial is currently recruiting to test the safety and efficacy of telitacicept against placebo in a cohort of 166 NMOSD patients (ClinicalTrials.gov Identifier: NCT03330418).

#### 4.3.3 Satralizumab

Satralizumab is a humanized monoclonal recycling antibody against the interleukin-6 (IL-6) receptor that is approved for the treatment of NMOSD in Canada, the USA, Japan, Switzerland, Europe and other countries by their respective regulatory authorities (113). By binding to the IL-6R, it reduces B cell-derived plasmablasts in the periphery and AQP4-Ab secretion by B cells. Both effects explain the therapeutic effect in NMOSD (105, 114).

In a randomized controlled trial involving 83 NMOSD patients (the Sakura-Sky trial, 70% positive to AQP4 Ab’s), sartralizumab was tested as an add-on therapy to conventional immunosuppressive treatment against placebo. The relapse rate of 20% for sartralizumab was significantly lower than that for placebo (43%). The percentage of patients who were free from relapse at 96 weeks were 78% (sartralizumab) and 59% (placebo).In the secondary endpoints, sartralizumab did not reduce pain and fatigue symptoms (115).One year later, results were available for a 95-patient NMOSD (the Sakura-Star) trial testing sartralizumab as monotherapy against placebo. With a relapse rate of 30%, sartralizumab was superior to 50% with placebo, although this significant result was not noted in the subgroup of seronegative patients. Among the seropositive participants, 77% had no relapse after 96 weeks with sartralizumab, whereas only 41% had no relapse with placebo. Again, no robust difference between the substances could be objectified with regard to pain and fatigue (116).

### 4.4 Bruton’s tyrosine kinase inhibitors

BTK inhibitors are a relatively new class of small molecules explored for utility in the treatment of MS. By inhibiting this tyrosine kinase, they have an effect on the innate immune system including microglial cells and monocytes/macrophages as well as on the adaptive immune response by modulating B cells proliferation and activation. Recent work found evidence that BTK inhibition drives myeloid cells into a regulatory phenotype, promoting myelin repair (40, 117). BTK inhibitors may impact the innate immune system through interactions with the surface receptors of the Toll-like group and modification of upstream proteins such as Mal/TIRAP or Toll-like receptor 13 d. In addition, inflammasomes such as the multi-protein complex NLRP3 are regarded as components of BTK signalling and hence may be downregulated by BTK inhibitors (118–122). BTK inhibitors are considered promising candidates for MS therapy, as they can cross the blood-brain barrier (123) and, through their additional effect on the innate immune system, they may also address compartmentalized inflammation, which is of particular importance in mediating brain damage and driving disability of MS (119, 124–127). 

#### 4.4.1 Iribrutinib

Iribrutinib, the first market-ready BTK inhibitor, was developed in 2007 and approved by the FDA in 2013 for the treatment of mantle cell lymphoma and chronic lymphocytic leukemia. Due to its irreversible and non-specific binding to cysteine residue-481 of the kinase domain, it interacts with other kinases such as EGFR, JAK3, HER2 and TEC (off-target effects). This results in drug side effects such as cardiac arrhythmias, diarrhea, bleeding, hypertension and arthralgia (118). New generations of BTK inhibitors, which are also being tested in MS studies, are designed to reduce these effects.

#### 4.4.2 Evobrutinib

Evobrutinib binds irreversibly to cysteine residue 481 of the kinase domain, but increases its selectivity to avoid off-target effects by additionally interacting with threonine residue 474 (128).

A double-blind, placebo- and comparator-controlled (dimethyl fumarate) phase 2 study of 267 RMS patients yielded complex results. The daily dose of 75 mg met the primary endpoint of cumulative total number of gadolinium-enhancing T1 lesions at weeks 12 to 24. Surprisingly, both the lower (25 mg) and higher (75 mg twice daily) dose of evobrutinib compared to the placebo group showed no significant difference with respect to the primary endpoint (129). The currently ongoing phase 3 evolution RMS 1 trial may shed a different light on the effect of the BTK inhibitor. In this trial, the agents evobrutinib and teriflunomide are being compared in a placebo-controlled manner in a planned population of 898 RMS patients (ClinicalTrials.gov identifier: NCT04338022 - first results expected in September 2023). According to current experience from studies in MS, an increased number of nasopharingitides and elevated liver enzymes are side effects of evobrutinib (129).

#### 4.4.3 Tolebrutinib

Tolebrutinib functions as a covalent irreversible BTK inhibitor that specifically binds to cysteine residue 481 of the kinase domain (130).

The results of a randomized, double-blind, placebo-controlled, 16-week phase 2 trial in 126 RMS or SPMS patients have been published in 2021 (120). A 4-week treatment with placebo at the start or end of the 16-week treatment was combined with a 12-week treatment with tolebrutinib at doses of 5, 15, 30 or 60 mg per day. The primary endpoint was the number of new gadolinium-enhancing lesions after 12 weeks of tolebrutinib treatment, which was achieved by all dose groups, while the strongest effect in the 60 mg group. In these patients, there was a relative reduction of new gadolinium-enhancing lesions of 85% compared to placebo. The observed mean number of lesions was 0.13 for tolebrutinib 60 mg versus 1.03 for placebo (120). Encouraged by the results, four phase III trials are currently underway: The GEMINI I/II twin trials recruited a total of 900 RMS patients with randomized to receive either tolebrutinib or teriflunomide (ClinicalTrials.gov identifier: NCT04410991 - first results are expected in August 2023). The HERCULES trial is testing tolebrutinib against placebo in a planned cohort of 1290 non-relapsing SPMS patients (ClinicalTrials.gov Identifier: NCT04411641 - first results expected in August 2024). 990 PPMS patients are planned to receive tolebrutinib or placebo in the ongoing PERSEUS trial (ClinicalTrials.gov Identifier: NCT04458051 - first results expected in August 2024).

Based on the study results to date, headache and questionable elevation of liver enzymes are potential side effects of tolebrutinib (120). In June 2022, the FDA temporarily placed a partial clinical hold on the trial in this regard (131).

#### 4.4.4 Fenebrutinib

Fenebrutinib is a non-covalent reversible BTK inhibitor that exerts its effect through hydrogen bonding with lysion-430, methionine-477 and apsartate-539 of the kinase domain. Due to the alternative mechanism of action, off-target effects may be avoided (118, 121).

Two phase III trials are currently underway. In the FENtrepid study, placebo-controlled fenebrutinib and ocrelizumabare being tested for their effect in a planned group of 946 PPMS patients (ClinicalTrials.gov Identifier: NCT04544449 - first results expected in October 2025). 734 RMS patients are being compared against the comparator teriflunomide in the FENhance trial (ClinicalTrials.gov Identifier: NCT04586023 - first results expected in October 2025).

Side effects observed in previous studies in rheumatoid arthritis, include nausea, headache, anemia and upper respiratory tract infections (121).

#### 4.4.5 Orelabrutinib

Orelabrutinib irreversibly binds to the kinase domain with exceptionally high selectivity and has been shown to have little binding to other kinases (132, 133).

A phase 2 trial of 160 RMS patients is currently underway, testing three different dose concentrations against placebo (ClinicalTrials.gov identifier: NCT04711148 - first results expected in July 2023).

From previous phase 1 trials, petechiae and headache are coming into focus as possible side effects (133).

#### 4.4.6 Remibrutinib

Remibrutinib is an orally administered irreversible covalently binding BTK inhibitor (127, 134).

Results from MS studies have not yet been presented, but the safety and efficacy profile of the compound have been investigated in a phase I study in healthy volunteers with and without atopic diathesis (134). Encouraged by the results, a phase III trial is currently taking place in which remibrutinib is being tested against the comparator teriflunomide in a collective of 800 RMS patients (ClinicalTrials.gov Identifier: NCT05147220/NCT05156281 - first results expected in October 2025).

## 5 Conclusion

In summary, important milestones in the drug therapy of MS have been achieved in the last 10 years. While the focus was initially on controlling acute relapses of MS, efforts are increasingly moving in the direction of establishing therapy options for progressive disease. With the CD20-depleting antibody ocrelizumab and the sphingosine receptor modulator siponimod, drugs for primary and secondary chronic MS are available for the first time. Great hopes are pinned on the class of Bruton’s tyrosine kinase inhibitors, which address not only the adaptive but also the innate immune system, which is thought to be responsible for the maintenance of chronic MS disease progression. The results the ongoing phase 3 trials with BTKi’s are eagerly awaited. Meanwhile, the understanding of the pathogenesis of MS, MOGAD and NMOSD continues to deepen. Even though the study situation on drug therapy options in NMOSD and MOGAD has not yet reached the breadth of those in MS, the fields of cytokine antagonists and CD19+ B cell depletors offer candidates for further therapeutic options. All in all, there seems to be reason for cautious optimism that the therapeutic successes of the last 10 years in the field of inflammatory autoimmune CNS diseases can be continued.

## Author contributions

Study concept and design: MF, RM, H-PH. Acquisition of data: MF, RM, H-PH. Analysis and interpretation of data: MF, MD, JM, RM, H-PH. Drafting of the manuscript: MF, MD, JM, RM, H-PH. Critical revision of the manuscript for important intellectual content: SM, PA, JM. Graphical support: HB. Study supervision: RM, H-PH.All authors contributed to the article and approved the submitted version.
