# Supplementary material for: B cell targeted therapies in inflammatory autoimmune disease of the central nervous system
Source: Front Immunol. 2023 Mar 9;14:1129906. doi: 10.3389/fimmu.2023.1129906 (PMC10034856; doi:10.3389/fimmu.2023.1129906)
Supplement: Supplementary file 1 [file Table_1.docx]

Supplementary Material

Therapeutic B cell depletion in inflammatory autoimmune disease of central nervous system

**Moritz J Furman, Sven G Meuth, Philipp Albrecht, Michael Dietrich, Heike Blum, Jan Mares^3^,Ron Milo, Hans-Peter Hartung***

*** Correspondence:** Hans-Peter Hartung: hartunghp@gmail.com

# Supplementary Table

**Table 1.** Key studies of B cell depleting therapies.

| **Trail** | **Primary end point and result** | | **Secondary end point and result** |
| --- | --- | --- | --- |
| ***CD20 antigen related drugs*** | | | |
| **Rituximab** | | | |
| Phase 1 (26 patients with RRMS), Bar-Or et al. 2008 | -Safety of 2 cycles of rituximab (adverse events, number of infusion-associated events, laboratory parameters, total number of Gd-enriching T1 lesions)  -> No serious adverse events were noted. | | -Development of gadolinium-absorbing t1 lesions or new t2 lesions after 72 weeks.  -> No new lesions after 72 weeks |
| Phase 2 (104 patients with RRMS), Hauser et al. 2008 (HERMES trail) | -Total count of gadolinium-enhancing lesions detected on magnetic resonance imaging scans of the brain at weeks 12, 16, 20, and 24  -> Rituximab superior to placebo | | -Proportion of patients with relapses  -Annualised relapse rate  -Absolute number of new gadolinium-positive T1 lesions in serial brain MRIs at weeks 12, 16, 20, 24  -Change in T2 lesion volume compared to baseline  -> Rituximab superior to placebo (but no reduction in long-term relapse rate) |
| Phase 2/3 (439 patients with PPMS), Hawker et al. 2009 (OLYMPUS trail) | - Time to confirmed disease progression (rise in Expanded Disability Status Scale sustained over 12 weeks)  -> Rituximab is not superior to placebo.  Exception: Rituximab shows advantage over placebo in young patients (<51 years) with inflammatory lesions | | -Change in T2 lesion volume  -Change in total brain volume  -> Rituximab leads to less T2 lesion load increase than placebo, whereas groups are identical in terms of brain volume change |
| Phase 2 (38 patients with aquaporin-4-Ak seropositivity), Tahara et al. 2020 (RIN-1 trail) | - Time until first relapse within 72 weeks  -> Rituximab is not superior to placebo. | | -Post-hoc analysis: 32% of patients receiving rituximab show a decrease in aquaporin-4-Ak titer  -> Exact mechanism of action of rituximab in NMOSD? |
| **Ocrelizumab** | | | |
| Phase 2 (220 patients with RRMS), Kappos et al. 2011 | | -Number of gadolinium-positive T1 lesions at weeks 12, 16, 20 and 24.  -> 600 mg and 2000 mg ocrelizumab groups are superior to placebo. 600 mg and 2000 mg groups appear to be equivalent. | -Annualized relapse rate  -Percentage of nonrelapsing patients  -change in absolute T2 lesion volume  -number ofnew gadolinium-positive T1 lesions between weeks 4 and 24  ->Ocrelizumab is superior to placebo in annulised relapse rate and number of new gadolinium-positive T1 lesions |
| Phase 3 (1656 patients with RRMS - 821 Opera I, 835 Opera II), Hauser et al. 2017 (OPERA I and II Trail) | | -Annualised relapse rate over 96 weeks  -> Ocrelizumab is superior to interferon-beta | Clinical endpoints:  -Disability progression manifest over 12 weeks.  -Multiple sclerosis functional composite scores  -Development of Short Form Health Survey-36 (SF-36) by week 96.  -Proportion of patients with EDSS greater than/equal to 2.0 who had no evidence of disease activity by week 96  MRI endpoints:  -average number of gadolinium-enhancing lesions T1 lesions W 24,48,96  -hypointense lesions on T1 W 24,48,96  Safety profile  -immunogenicity of ocrelizumab  -safety profile of ocrelizumab.  -> Ocrelizumab is superior to interferon-beta in most secondary endpoints related to clinical and mr-tomographic outcome. However, there was no superiority in the items improvement in multiple sclerosis function score including SF-36 score and total brain volume loss. In addition, a higher number of infusion reactions and twice the absolute number of neoplasia manifestations occurred with ocrelizumab compared to interferon-beta (4 versus 2). |
| Phase 3 (732 patients with PPMS), Montalban et al. 2017 (ORATIO trail) | | -Percentage of patients with disability progression confirmed at 12 weeks  ->Ocrelizumab is superior to placebo | clinical endpoints:  -percentage of patients with disability progression confirmed at 24 weeks  -change in performance on the timed 25-foot walk from baseline to week 120  -change in the Physical Component Summary score of the Medical Outcomes Study 36-Item Short-Form Health Survey (SF-36)  MRI endpoints:  -change in the total volume of brain lesions on T2 -weighted MRI from baseline to week 120  -change in brain volume from week 24 to week 120  -mean number of new or enlarging hyperintense lesions on T2-weighted images from baseline to week 120  Safety profile:  -immunogenicity of ocrelizumab  -safety profile of ocrelizumab.  -> Ocrelizumab achieves all secondary endpoints except for an improvement in the 36-item Short-Form Health Survey (SF-36). Compared to the placebo group, more infusion reactions up to the intermediate category and an increased incidence of neoplasia are observed with ocrelizumab. |
| Follow-up study Opera I and II until 5 years after start (RRMS), Hauser et al. 2020 | | -Period to onset of 24-week confirmed disability Progression after 5 years  -Gadolinium-enhanced and new/enhancing T2 lesions after 5 years  -Percentage brain volume change after 5 years  -> Compared to patients switched from interferon-beta to ocrelizumab, earlier and continuous ocrelizumab therapy for up to 5 years provided a sustained benefit in clinical and MRI measures of disease progression. No new safety findings emerged. |  |
| Follow-up study Oratio until 6.5 years after start (PPMS), Wolinsky et al. 2019 | | -24-week confirmed disability progression after 6.5 years  -confirmed disability progression 24 on the 9-Hole PegTest after 6.5 years  -Safety profile after 6.5 years  -> After 6.5 study-years' follow-up, patients who initiated ocrelizumab 3–5 years earlier accrued less disability progression compared with patients switching from placebo. |  |
| Phase 3b (576 patients with RRMS and a clinical or radiological objectified relapse under one or more DMT for at least 6 months), Weinstock-Guttman et al. 2022 (CHORDS trail) | | -Absence of evidence of disease activity called NEDA (= absence of protocol-defined relapse, confirmed disability progression, T1 Gd-enhancing lesions and new/enhancing T2 lesions).  -> Over 96 weeks, 48.1% of patients achieved NEDA | Within 24, 48 or 96 weeks free of any of the following:  -absence of a relapse defined according to the protocol  -A confirmed progression of disability  -T1 Gd-enhancing lesions  -new/enhancing T2 lesions  -> Free of protocol-defined relapse (89.6%), confirmed disability progression (89.6%), T1 Gd-enlarging lesions (95.5%) and new/enlarging T2 lesions (59.5%) at week 96. |
| Phase 3b (680 patients with RRMS and a clinical or radiological objectified relapse under one or more DMT for at least 6 months), Vermersch et al. 2022 (CASTING trail) | | -Absence of evidence of disease activity called NEDA (= absence of protocol-defined relapse, confirmed disability progression, T1 Gd-enhancing lesions and new/enhancing T2 lesions).  -> Over 96 weeks, 74.8 % of patients achieved NEDA | -No disease activity at 96 weeks: 76.3%.  -24 weeks no increase in EDSS score from baseline by at least 1.0 point by week 96: 88.4%.  -Almost complete suppression of MRI lesion activity was observed, with most patients having no contrast-enhancing lesions from week 8 to 96 and no new and/or enlarging T2-weighted hyperintense lesions (new/enhancing T2 lesions after week 96: 14)  -The total T2-weighted hyperintense lesion volume decreased over time with a mean percentage change of -8.5%.  -The normalised brain volume decreased over time with a mean percentage change from baseline of -0.81% at week 96. |
| **Ofatumumab** | | | |
| Phase 2 (232 RRMS patients with and without prior MS therapy), Bar-Or et al. 2018 (MIRROR trail) | | - Cumulative number of new gadolinium-enhancing lesions (based on T1-weighted MRI scans at weeks 4, 8 and 12) at week 12.  -> Reduction in the mean rate of new GdE lesions by 65% of all ofatumumab groups compared to placebo | -Cumulative number of new GdE lesions at week 24.  -Cumulative number and total volume of new plus persistent GdE lesions (week 12,24).  -new and/or newly enlarging T2 lesions and T1 hypointense lesions (week 12,24)  -Proportion of patients who were relapse-free from week 0 to 12.  -EDSS, MSFC (Multiple Sclerosis Functional Composite) and MFIS (Modified Fatigue Impact Scale) scores  -> Ofatumumab superior to placebo in radiological parameters, no significant difference in clinical parameters |
| Phase 3 (1882 RRMS or SPMS patients), Hauser et al. 2020 (ASCLEPIOS I + II trail) | | -Adjusted annualised relapse rate 0.11/0.10 with ofatumumab and 0.22/0.25 with teriflunomide (Asclepios I/II)  -> Ofatumumab superior to teriflunomide | -Confirmed worsening of disability after 3/6 months.  -After 6 months, confirmed improvement of disability  -Number of new or enlarging lesions on T2-weighted MRI scan per year and annual rate of brain volume loss.  -Concentration of neurofilament light chain in serum after month 3 and beyond.  -relationship between neurofilament light chain concentration at baseline and formation of new or enlarging lesions on T2-weighted MRI.  -> In terms of clinical parameters, ofatumumab slowed the deterioration of disability, whereas no significant difference in disability improvement was found between the two drugs. Radiological endpoints were met with the exception of the annual rate of brain volume loss. Ofatumumab showed lower serum neurofilament light chain concentrations at months 3, 12 and 24 compared to the teriflunomide group. |
| Phase 3b (1969 RRMS patients who were either already treated with ofatumumab (n=1292) or switched from teriflunomide to ofatumumab (n=677)), Hauser et al. 2022 (ALITHIOS trail) | | - Safety profile from cumulative data of ofatumumab continuous treatment and patients switched from teriflunomide.  -> Good tolerability and no new safety signals compared to Asklepios I/II |  |
| **Ublituximab** | | | |
| Phase 2 (48 RRMS patients with specific pre-treatment drug requirements., randomisation in a 3:1 ratio of utilizumab concentration/infusion rate vs. placebo), Fox et al. 2021 | | - Proportion of patients treated with ublituximab who achieved ⩾95% peripheral CD19+ B-cell depletion from baseline with at week 4.  -> 100 % B-cell depletion in the serum of the utilizumab groups | -Gadolinium-enhancing T1-weighted lesions.  -change in T2-weighted lesion volume.  -number of new or enhancing T2 lesions on MRI -annualised relapse rate (ARR)  -proportion of relapse-free patients.  -frequency of infusion-associated events at day 15 and week 24 as a function of dose and infusion rate.  -> Radiological endpoints were met, there was no positive modification of disability, the number of infusion-related adverse events was independent of infusion rate and dose |
| Phase 3 (1094 RRMS patients), Steinman et al. 2022 (ULTIMATE I/II trail) | | - Anualised relapse rate ublituzimab versus teriflunomide  -> Adjusted annualised relapse rate over a 96-week period 0.08/0.09 in the ublituximab group I/II versus 0.19/0.18 in the teriflunomide group I/II | -mean total number of gadolinium-enhancing lesions per T1-weighted MRI scan.  -mean total number of new or enhancing hyperintense lesions per T2-weighted MRI-.  -percentage change in brain volume  -worsening of disability at 12 and 24 weeks  -absence of disease activity at 96 weeks  -median number of CD19+ B cells 24 hours after the first dose.  -> Ublituximab leads to fewer brain lesions on MRI than teriflunomide and to effective CD19 depletion but not to a significantly lower risk of worsening disability. |
| Phase 1 (5 Patients with aquaporin-4-immunoglobulin G (IgG)-seropositive neuromyelitis optica spectrum disorder (NMOSD)), Mealy et al. 2019 | | - Safety of the additional single dose of ublituximab  -> No occurrence of serious adverse events | -Change in Expanded Disability Status Scale (EDSS).  -number of B cells after single dose  -> EDSS median score decreased from 6.5 at admission to 4.0 at 90-day follow-up. Two patients did not achieve complete B-cell depletion |
| ***CD19 antigen directed drugs*** | | | |
| **Inebilizumab** | | | |
| Phase 1 (28 RRMS patients), Agius et al. 2017 | | - Safety and tolerability of ascending intravenous (30, 100, 600 mg) and subcutaneous (60, 300 mg) doses of inebilizumab  -> Good tolerance. Most common side effects: nasopharyngitis (24 %), upper respiratory tract infections (19 %), urinary tract infections (14 %), injection-associated side effects such as fever and tachycardia. | -Pharmacokinetics and -dynamics  -number of new gadolinium (Gd)-enhancing lesions and the number of new or newly enlarging T2 lesions.  -Recurrences  -EDSS scores  -Tetanus titre  -> Inebilizumab fulfils MRI parameters, whereas there is no change in clinical parameters or in tetanus titer. |
| Phase 2/3 (230 seropositive or -negative NMOSD patients with at least one emergency therapy due to relapse within the last year or with 2 emergency therapies within the last 2 years), Cree et al. 2019 (N-MOmentum trail) | | - Time to occurrence of NMOSD attack over period of 197 days  -> Within the observation time, 12% of the inebilizumab group relapsed, compared to 39% of the placebo group. | -Deterioration of EDSS score from baseline.  -change from baseline in the low-contrast visual acuity binocular score.  -Cumulative total number of active MRI lesions (new gadolinium-enhancing lesions or new or enlarging T2 lesions measured in the optic nerve, brain, brainstem and spinal cord).  -Number of NMOSD-related inpatient hospitalisations  -> Inebilizumab group superior to placebo in terms of worsening EDSS score, new MRI lesions and cumulative hospitalisations - no difference in terms of contrast vision. |
| Phase 3b (75 aquaporin-positive NMOSD patients treated with inebilizumab for a total of 4 years), Rensel et al. 2022 | | - Efficacy and safety of inebilizumab after 4 years of use  -> Good efficacy of inebilizumab (83% of users were attack-free after 4 years) without new safety warnings |  |
| ***Cytokine antagonists*** | | | |
| **Atacicept** | | | |
| Phase 2 (34 patients with unilateral optic neuritis without a diagnosis of MS), Sergott et al. 2015 (ATON trail) | | -Safety and efficacy of atacicept compared to placebo over 36 weeks  -> Early study termination due to higher conversion of atacicept arm to final MS diagnosis compared to placebo arm (35.3% versus 17.6%). At the same time, however, there was less axonal retinal thickness loss in the atacicept group compared to the placebo group (-8.6 µm versus -17.3 µm). |  |
| Phase 2 (255 patients with RRMS randomised in a 1:1:1:1 ratio to 3 atacicept and 1 placebo group), Kappos et al. 2014 (ATAMS trail) | | - Change in the average number of gadolinium-enhancing lesions in T1-weighted MRI per patient.  -> Early study termination due to increased annualised relapse rates in all atacicept groups compared to placebo group with similar number of gadolinium-enhancing T1 lesions. |  |
| **Telitacicept** | | | |
| Phase 2 (8 patients with recurrent NMOSD attacks), Ding et al. 2022 | | - Time of first relapse after inclusion in the study within 48 weeks  -> Two patients (25 %) relapsed and five patients (63 %) remained relapse-free after 48 weeks of treatment. The relapse of the two patients occurred at a longer relapse interval compared to the interval before inclusion in the study. | -changes in EDSS score  -Score, Hauser Ambulation Index  -Number of lesions on MRI  -RNFL thickness on OCT  -Latency and amplitude of VEP  AQP4 antibody titre -stable  -> Clinically discrete improvement with constant functional diagnostic findings and slight radiological deterioration |
| **Sartralizumab** | | | |
| Phase 3 (83 patients with existing baseline therapy for NMOSD were randomised in a 1:1 ratio to add-on therapy with sartralizumab or placebo), Yamamura et al. 2019 (Sakura-Sky trial) | | - First protocol-defined relapse in a time-to-event analysis (average 107 weeks of treatment).  -> Sartralizumab as add-on therapy superior to placebo (20% vs. 43%). The percentage of patients who were free from relapse at 96 weeks were 78% (sartralizumab) and 59% (placebo). | - Among other things, change  -the visual analogue scale (VAS) for pain (on a scale of 0 to 100, with higher scores indicating more pain)  -the Functional Assessment of Chronic Illness Therapy-Fatigue (FACIT-F) score (on a scale of 0 to 52, with lower scores indicating more fatigue)  from baseline to week 24.  -> No significant changes compared to placebo. |
| Phase 3 (95 NMOSD patients randomised 2:1 to monotherapy placebo vs. sartralizumab), Traboulsee et al. 2020 (Sakura-Star trail) | | - Time until the first relapse defined according to the protocol  -> Sartralizumab superior to placebo with relapse rate of 30% versus 50%. Better results for seropositive patients than for seronegative ones. Among the seropositive participants, 77% had no relapse after 96 weeks with sartralizumab, whereas only 41% had no relapse with placebo. | -Change in pain score on the Visual Analogue Scale (VAS) by 24 weeks.  -Change in fatigue score on the Functional Assessment of Chronic Illness Therapy (FACIT)24 from baseline to week 24.  -proportion of relapse-free patients  -annualised relapse rate  -Monitoring of adverse events  -> No significant difference in pain and fatigue symptoms between groups. |
| ***Bruton's Tyrosine Kinase (BTK) inhibitors*** | | | |
| **Tolebrutinib** | | | |
| Phase 2b (126 RRMS or SPMS patients), Reich et al. 2021 | | -Number of new gadolinium-enriching lesions after 12 weeks of tolebrutinib treatment  -> Maximum effect was 60 mg of tolebrutinib per day. Relative reduction of new gadolinium-enhancing lesions of 85% compared to placebo. The observed mean number of lesions was 0.13 for tolebrutinib 60 mg vs. 1.03 for placebo. | -number of new or enlarging T2 lesions  -Total number of gadolinium- lesions  -adverse events  -serious adverse events  -> Partial fulfilment of radiological criteria (new enlarging T2 lesions), headache as undesirable side effect, questionable also alanine aminotransferase elevation. |
| **Evobrutinib** | | | |
| Phase 2 (267 patients with RRMS), Montalban et al. 2019 | | -The cumulative total number of gadolinium-enhancing lesions identified on T1-weighted MRI at weeks 12 to 24.  ->Only the evobrutinib 75 mg once daily group showed a significant difference with respect to the primary endpoint compared to the other evobrutinib doses and placebo. | -annualised relapse rate  -relapse-free status  -change in EDSS score at week 24 from baseline.  -safety  -> The clinical secondary endpoints were not reached by any dose of evobrutinib. Side effects included transiently elevated liver enzymes and nasopharingitis. |
| **Fenebrutinib** | | | |
| Phase 3 (In the FENtrepid study, placebo-controlled fenebrutinib and ocrelizumabare being tested for their effect in a planned group of 946 PPMS patients) | | currently underway | currently underway |
| **Orelabrutinib** | | | |
| Phase 2 (A phase 2 trial of 160 RMS patients is currently underway, testing three different dose concentrations against placebo) | | currently underway | currently underway |
| **Remibrutinib** | | | |
| Phase 3 (a phase III trial is currently taking place in which remibrutinib is being tested against the comparator teriflunomide in a collective of 800 RMS patients) | | currently underway | currently underway |
